# Association between E-Cigarette Use Behaviors and Anxiety/Depression among Black/African American Adults Based on Sexual Identity

**DOI:** 10.3390/ijerph20032078

**Published:** 2023-01-23

**Authors:** David Adzrago, Melissa B. Harrell, Kayo Fujimoto, Antwan Jones, J. Michael Wilkerson

**Affiliations:** 1 Center for Health Promotion and Prevention Research, School of Public Health, The University of Texas Health Science Center at Houston (UTHealth), Houston, TX 77030, USA; 2 Department of Epidemiology, Human Genetics & Environmental Sciences, UTHealth Austin School of Public Health, Austin, TX 78701, USA; 3 Department of Sociology, and Department of Epidemiology, The George Washington University, Washington, DC 20052, USA

**Keywords:** electronic cigarettes, tobacco use, mental health, minority, identity

## Abstract

Limited studies have examined disparities in e-cigarette use among Black/African American adults by sexual identity and whether the relationship between symptoms of anxiety/depression and e-cigarette use varies by sexual identity. We examined the association between e-cigarette use behaviors (never, former, and current use) and anxiety/depression among a nationally representative sample of Black/African American adults who identified as a sexual minority (lesbian/gay, bisexual, and others) or heterosexual individuals. We combined cross-sectional data from the 2011 to 2020 Health Information National Trends Survey (n = 6267), which is a nationally representative data set. We computed weighted e-cigarette use prevalence and multinomial logistic regression results (never use compared with former and current use, respectively). Among Blacks/African Americans, a larger percentage of sexual minority individuals compared with heterosexual individuals reported former and current e-cigarette use. Among sexual minorities, lesbian/gay individuals reported higher former e-cigarette use, whereas bisexual individuals reported higher current e-cigarette use. Among sexual minority individuals, moderate symptoms of anxiety/depression, compared with no symptoms of anxiety/depression, were associated with a higher likelihood of former e-cigarette use. Among heterosexuals, moderate symptoms of anxiety/depression were also associated with a higher likelihood of former e-cigarette use, while mild and severe symptoms of anxiety/depression were associated with current e-cigarette use compared with no symptoms of anxiety/depression. The intersection between sexual identity and anxiety/depression influenced e-cigarette use behaviors in different ways among Black/African Americans. The findings reinforce the heterogeneity within the Black/African American population, indicating the dangers of not considering subgroup differences as a standard part of public health research practice.

## 1. Introduction

While the prevalence of e-cigarette use in the United States (U.S.) was reported to be lower for Black/African American adults than for White American adults [1,2,3], the prevalence within the Black/African American population may be lower or higher depending on their sexual identity (i.e., self-identity as a sexual minority or heterosexual). Between 2014 and 2019, for example, there were 5.6–10% former and 1.9–3.4% current Black/African American adult e-cigarette users compared with 10.3–13.2% former and 4.5–5.1% current White American adult e-cigarette users [1,2,3]. However, e-cigarette use is higher among sexual minority individuals (i.e., self-identified as lesbian, gay, or bisexual (LGB)) than among heterosexual individuals [4,5,6].

Former (36.5%) and current (22.3%) e-cigarette use was higher for sexual minority individuals than for heterosexual individuals (former use = 18.1% and current use = 19.7%) [7]. This e-cigarette use prevalence may imply that Black/African American adults who are sexual minority individuals are more likely to use e-cigarettes than their heterosexual counterparts. Hence, the notion that e-cigarette use is lower for all Black/African American adults may require more information because this behavior can vary by their sexual identity. Nonetheless, limited studies examined sexual identity disparities in e-cigarette use among the Black/African American adult-only population to identify the most-at-risk groups. The dearth of literature on sexual identity disparities in e-cigarette use among Black/African Americans may also be due to a lack of studies evaluating this behavior among the Black/African-American-only population.

Black/African American and LGB individuals have the highest health problems related to using any tobacco product, such as e-cigarettes and others (cigarettes, cigars, cigarillos/little cigars, and hookahs) [7,8,9,10,11]. As with other tobacco products, e-cigarettes contain nicotine and other chemical toxins that are addictive and harmful to consume [12,13,14,15,16,17]. The lack of literature on e-cigarette use among Black/African American and LGB adults, coupled with the high health risk of tobacco use, such as e-cigarette use, requires an assessment of the likelihood of e-cigarette use among this population.

Mental health disorder symptoms, particularly anxiety/depression, are predominant among Black/African American and LGB adults [18,19]. Previous general population studies suggest that anxiety/depression symptoms may explain e-cigarette use behavior among Black/African American adults based on sexual identity [7,20,21,22,23,24,25]. These previous studies established that LGB individuals were more likely to use e-cigarettes than heterosexual individuals [4,6,26,27], particularly LGB individuals experiencing depression symptoms [7,21,22,28]. Likewise, former or current e-cigarette use was more likely to occur among Black/African American individuals with symptoms of depression [29]. However, none of these studies looked specifically at e-cigarette use among Black/African American adults or sexual identity disparities in this population, nor whether the relationship between symptoms of anxiety/depression and e-cigarette use varied by sexual identity status. Instead, these studies only included race/ethnicity as a covariate in their analyses.

Previous studies showed that differences in e-cigarette use among Black/African American adults based on their sexual identity could be explained by age, sex, level of education, income, U.S. census region, and general health status [7,27,30]. According to Hoffman et al. [7], sexual minority females and males, including gay/lesbian and bisexual females, were more likely to use e-cigarettes than heterosexual females. This study also found that young adults, males, and individuals with lower education and income were more likely to use e-cigarettes. Similarly, Delnevo et al. [2] reported that individuals residing in the Southern, Midwestern, or Northeastern regions of the U.S. were more likely to use e-cigarettes compared with their Western counterparts. Additionally, it was found in a national study that individuals who perceived their general health as good were less likely to use e-cigarettes than those who perceived their general health as fair or poor [30]. Examining these sociodemographic characteristics in relation to e-cigarette use among Black/African American adults who identified as a sexual minority or heterosexual individuals can thus help to determine inter- and intragroup disparities for tailored e-cigarette use reduction and cessation interventions.

This analysis sought to bridge the gap in the literature and expand on it by assessing the association between e-cigarette use behaviors (i.e., ever, former, and current use) and anxiety/depression symptoms among Black/African American adults to examine whether this relationship varies by sexual identity. We examined e-cigarette use behaviors among a nationally representative sample of Black/African American adults to generalize our findings to this population for more extensive tailored interventions. We particularly estimated the prevalence of e-cigarette use among Black/African American adults who identified as sexual minority individuals compared with heterosexual individuals based on anxiety/depression symptoms and sociodemographic characteristics, such as age, sex, level of education, income, general health status, and U.S. census region. Additionally, we assessed the association between e-cigarette use and anxiety/depression symptoms among Black/African American adults, stratified by sexual identity and adjusting for the sociodemographic characteristics.

## 2. Materials and Methods

### 2.1. Study Design

We used the Health Information National Trends Survey (HINTS) dataset, which is an annual cross-sectional survey. HINTS was established in 2002/2003 by the U.S. Department of Health and Human Services and is administered annually [31,32,33]. The HINTS was conducted using computer-assisted telephone interviews (CATI) from 2002/2003 to 2008. The HINTS in 2008 applied the mailing method as well. The subsequent HINTSs after 2008 were conducted using a single-mode mail survey or mailing method. Of note, the 2009 survey was undertaken only in Puerto Rico. The remaining HINTSs were conducted annually from 2011 to 2020. As a result of the consistency in their data collection methods and data recency, the datasets from these subsequent HINTSs from 2011 were included in our study.

Secondary data analysis is exempt from Institutional Review Board (IRB) review under federal regulations protecting human subjects [34]. However, to comply with The University of Texas Health Science Center at Houston (UTHealth) research policies and ethics, we obtained exempt approval for our data analysis from the UTHealth IRB.

### 2.2. Population/Sample

The HINTS is a survey of a nationally representative sample of civilian, noninstitutionalized U.S. adults aged 18 years and older to assess their tobacco use behavior, including e-cigarettes, and mental health disorder symptoms [32,33]. The HINTS also assesses participants’ sociodemographic characteristics, such as sexual identity and racial characteristics. The sampling strategies in the HINTS involved two-stage designs [33]. First, a stratified equal probability sample of addresses is chosen from two strata based on the minority population density (i.e., high versus low). In the final stage of this design, an adult is randomly selected from each of the chosen household addresses.

### 2.3. Data Sources and Inclusion/Exclusion Criteria

The HINTS is a publicly available dataset that includes de-identified data. Because the datasets contain information on Black/African American adults, we pooled data for these adults from the 2011 to 2020 surveys for our analysis and research questions [33]. The total sample size of Black/African American adults in these HINTSs was, not considering the missing data, 5389 out of a total sample size of 36,017. E-cigarette use did not significantly vary by the survey years (*p* = 0.1854), and thus, combining the datasets was relevant to achieving accurate associations and is an analytical method recommended by the HINTS [31,33].

### 2.4. Measures

For this study, the dependent variable was e-cigarette use behaviors, which included never, former, and current use. The questions that measured this variable included asking the participants whether they had ever used an e-cigarette, even once or twice (yes/no), and if they had, whether they now use an e-cigarette every day, some days, or not at all. The categories included never use if the participant responded no use to the first question, former use if the participant responded yes to the first question but no to the second question, and current use if the participant responded yes to the second question. As a result, the three categories were mutually exclusive, and the likelihood of being in one category versus another was analyzed.

The independent variables included anxiety/depression and sexual identity. Anxiety/depression status was based on four categories derived from the total scores of the Patient Health Questionnaire-4 (PHQ-4) [35,36]. The PHQ-4 is a four-question instrument ((1) feeling nervous, anxious, or on edge; (2) not being able to stop or control worrying; (3) feeling down, depressed, or hopeless; (4) little interest or pleasure in doing things) with a four-point Likert scale (1 = not at all, 2 = several days, 3 = more than half the days, and 4 = nearly every day) [35,36]. The total scores range from 0 to 12 (0–2 = normal/no anxiety/depression; 3–5 = mild; 6–8 = moderate; and 9–12 = severe) [35,36].

In the HINTS, participants were asked whether their sexual identity was heterosexual/straight, lesbian, gay, bisexual, or something else. Lesbian, gay, bisexual, and other groups were considered sexual minority groups, whereas heterosexual/straight was considered the heterosexual group.

The covariates included sociodemographic characteristics that were shown in previous studies to be associated with e-cigarette use behaviors. As a result, these variables were included as adjusted variables in our analysis. These variables included age (18–25, 26–34, 35–49, 50–64, 65 or more years), biological sex (female or male), level of education completed (less than high school, high school graduate, some college, or college graduate or higher), income (less than $20,000, $20,000 to <$35,000, $35,000 to <$50,000, $50,000 to <$75,000, or $75,000 or more), and U.S. census region (groupings of the U.S.’s 50 states and the District of Columbia into four subregions (Northeast, Midwest, West, or South) for the presentation of census data). The analysis also included participants’ general health status, which was determined by whether they considered their general health to be excellent, very good, good, fair, or poor.

### 2.5. Data Analysis

The HINTS sampling weights were used in the analyses to provide nationally representative results while accounting for nonresponse and noncoverage biases, as well as stratified probability sampling designs and clustering of the sampling units [31,33]. We calibrated the sampling weight for the ten years of pooled data to estimate the average population results based on those ten years and adjust for the years’ effects [33]. The analysis of Black/African American adults in the pooled dataset was conducted using the svy subpop command in STATA. Version 16.1 of the STATA software was used for the data analysis [37].

Descriptive and bivariate analyses were performed to estimate the prevalence of and differences in e-cigarette use behaviors based on sociodemographic characteristics and anxiety/depression among Black/African American adults who identified with heterosexual and sexual minority groups (Table 1). The bivariate analysis was computed using chi-squared tests with a *p*-value of 0.05 and a 2-sided inference test. Following that, a multinomial logistic regression analysis was performed to assess the association between e-cigarette use and anxiety/depression symptoms among Black/African American adults based on sexual identity, adjusting for sociodemographic factors (Table 2). The multinomial logistic regression analysis results were presented in the form of a relative risk ratio (RRR) with 95% confidence intervals (CIs) at a *p* < 0.05 for the 2-sided inference test.

As a preliminary analysis, we undertook the following statistical analyses and presented their results in supplemental files before collapsing sexual identity into heterosexual and sexual minority groups. We conducted descriptive statistics first to determine the prevalence of e-cigarette use behaviors by the sexual identity groups (heterosexual, lesbian, gay, bisexual, and other) among the Black/African American adults (Figure S1). Next, the prevalence of e-cigarette use behaviors by anxiety/depression based on sexual identity groups among the Black/African American adults was estimated (Figure S2). The results of these two analyses were presented using bar graphs. Furthermore, we used an unadjusted multinomial logistic regression model to examine the association between e-cigarette use behaviors and sexual identity groups (heterosexual, lesbian, gay, bisexual, and other) among the Black/African American adults (Table S1).

Missing observations for the variables were addressed using multiple imputations via chained equations to increase the statistical power [38,39,40,41]. There were 14.01% missing observations and the imputation resulted in 6267 samples for the analysis. The multicollinearity among the independent variables was examined based on the inflation factor (VIF) to identify significantly correlated (VIF value > 10) variables; the mean VIF of 1.13 suggested no issues of collinearity that would have resulted in unreliable statistical inferences about the results [42].

## 3. Results

### 3.1. Descriptive Statistics

The results of the descriptive analysis are displayed in Table 1. Overall, 32.03% of the Black/African American heterosexual individuals were aged 50–64 years; 57.54% were female; 30.35% had some college education; 34.37% had a total family annual income of less than $20,000; 21.01% had fair or poor general health; 54.15% resided in the South; and 16.83%, 7.26%, and 7.00% had experienced symptoms of mild, moderate, and severe anxiety/depression, respectively. Meanwhile, among the Black/African American sexual minority individuals, 26.40% were aged 35–49 years; 50.65% were male; 29.76% had some college education; 48.91% had a total family annual income of less than $20,000; 27.74% had fair or poor general health; 49.22% resided in the South; and 27.56%, 15.69%, and 14.12% had experienced symptoms of mild, moderate, and severe anxiety/depression, respectively.

### 3.2. Bivariate Differences in E-cigarette use Behaviors by Sociodemographic and Anxiety/Depression Factors

There were statistically significant differences in e-cigarette use behaviors by sexual identity (Table 1). Further, when e-cigarette use behaviors were stratified by sexual identity, significant differences were revealed by age, sex, level of education, and anxiety/depression symptoms. Specifically, a larger percentage of sexual minority individuals, compared with heterosexual individuals, reported former (23.53% vs. 10.19%) and current (5.78% vs. 3.65%) use of e-cigarettes. Particularly, as shown in Figure S1, lesbian/gay individuals reported the highest percentage of former e-cigarette use, whereas bisexual individuals reported the highest percentage of current e-cigarette use. As displayed in Figure S2, former or current use of e-cigarettes was common among both heterosexual and sexual minorities, as well as among those who did and did not have symptoms of anxiety/depression.

As shown in Table 1, sexual minority individuals aged 35–49 years (42.95 %) reported the highest prevalence of former e-cigarette use, while those aged 26–34 years (10.71%) reported the highest prevalence of current e-cigarette use. No bivariate differences in e-cigarette use behaviors by sex, level of education, total family annual income, general health status, U.S. census region, and anxiety/depression symptoms were observed among sexual minority individuals. In contrast, most heterosexual individuals aged 26–34 years (17.98%) reported former e-cigarette use, whereas those aged 18–25 years (8.52%) reported the highest prevalence of current e-cigarette use. Males reported higher former (12.55% vs. 8.45%) and current (4.98% vs. 2.66%) e-cigarette use than females. Those who reported higher former e-cigarette use had some college education (13.63%), while current e-cigarette users had high school education (5.55%). While the former e-cigarette users had the highest prevalence of moderate anxiety/depression (17.99%), current e-cigarette users had the highest prevalence of symptoms of severe anxiety/depression (10.13%). We did not observe bivariate differences in e-cigarette use behaviors by total family annual income, general health status, and U.S. census region among heterosexual individuals.

### 3.3. Multinomial Logistic Regression Analysis of E-Cigarette Use Behaviors and Their Risk Factors

The crude relative risk ratios of former e-cigarette use were higher for lesbian/gay and bisexual individuals compared with heterosexual individuals (Table S1). Bisexual individuals, compared with heterosexual individuals, were more likely to be currently using e-cigarettes.

The adjusted multinomial logistic regression analysis results stratified by sexual identity are shown in Table 2. Among heterosexual individuals, those aged 50 years or older were less likely to be former or current e-cigarette users compared with those aged 18–25 years. Females, compared with males, were less likely to be former or current e-cigarette users. Individuals who had some college education (compared with less than a high school education) were more likely to be former e-cigarette users. Those with a total family annual income of $35,000 to <$50,000 (compared with less than $20,000) were less likely to be former or current e-cigarette users. While symptoms of moderate anxiety/depression were associated with a higher likelihood of former e-cigarette use (RRR = 2.10; 95% CI = 1.31, 3.38), compared with no symptoms of anxiety/depression, symptoms of mild (RRR = 2.89; 95% CI = 1.34, 6.27) and severe (RRR = 5.44; 95% CI = 2.23, 13.23) anxiety/depression were associated with current e-cigarette use compared with no symptoms of anxiety/depression. Among sexual minority individuals, those who were aged 35–49 years were more likely to be former e-cigarette users, whereas those aged 50 years older were less likely compared with those aged 18–25 years. Having a total family annual income of $50,000 or more, compared with less than $20,000, was associated with a higher likelihood of former e-cigarette use. Those who experienced symptoms of moderate anxiety/depression, compared with no symptoms of anxiety/depression, had a higher likelihood of former e-cigarette use (RRR = 4.53; 95% CI = 1.38, 14.82).

## 4. Discussion

This population-level analysis made a novel contribution to the Black/African American-focused research by examining e-cigarette use behaviors and their risk factors among Black/African American adults based on their sexual identity. We found that sexual minority individuals had twice the prevalence of former and current e-cigarette use (versus never users) compared with heterosexual individuals. Specifically, lesbian/gay and bisexual individuals, compared with heterosexual individuals, were at least threefold more likely to be former e-cigarette users. Bisexual individuals had nearly a threefold risk of current daily or someday e-cigarette use (versus never use). These findings are consistent with previously published studies that showed that sexual minority persons, relative to heterosexual persons, are at disproportionately higher risk of e-cigarette use [4,5,6,7]. While the previous studies examined e-cigarette use in the general U.S. population, our study is the first national U.S. study to examine e-cigarette use behaviors by sexual identity among Black/African American adults, suggesting the need to consider sexual identity subpopulations in health and public health research. The findings imply that Black/African American adults may be at higher risk of e-cigarette use than previously reported given their sexual identity subgroups. It is imperative to consider that Black/African American adults are heterogeneous, and targeting them as a single group with the same tobacco control approach is likely to be inadequate to address tobacco-use-associated health disparities.

Our findings are in line with previous studies, which, although not conducted exclusively among Black/African American adults, showed that younger age is associated with a higher risk of e-cigarette use [6,27,29,30,43,44]. We found that heterosexual persons 50 years or older, relative to those aged 18–34 years, were less likely to be former and current e-cigarette users (versus never users). While sexual minority persons 50 years or older (versus 18–34 years) were less likely to be former e-cigarette users, those aged 35–49 years were more likely to be former e-cigarette users. However, there was no statistically significant difference in current e-cigarette use between sexual minority persons aged 18–34 years and those aged 35 years or older. These results augment the literature by including Black/African American subgroup analysis by sexual identity and age. Our findings imply within-group differences in e-cigarette use behaviors among Black/African American adults; certain groups of this population are more or less likely to be either former or current daily/someday e-cigarette users.

Compared with heterosexual and sexual minority males, females were less likely to be former or current e-cigarette users; however, the difference in former or current e-cigarette use among the sexual minority females was not statistically significant. These findings are similar to those reported in earlier studies that showed that males are, in general, more likely to use tobacco products, including e-cigarettes [6,7,45]. The male/female differences might be due to the overall higher health concerns or beliefs and a lower likelihood of engaging in health-compromising behaviors (e.g., tobacco use) among females than among males [46,47]. A possible reason for the non-statistically significant differences in e-cigarette use between male and female sexual minority persons might be due to the evidence that tobacco use is different among young sexual minority persons, but the use becomes similar as these persons transition to adulthood [48,49]. These males and females become homogenized in behaviors as they reach adulthood because they experience similar minority stresses and engage in similar coping behaviors, including tobacco use [6,45,48,50]. However, future longitudinal studies may better delineate the patterns of e-cigarette use and racial minority experiences in adolescence and transition to adulthood based on sexual and gender identity among Black/African Americans.

Similar to previous studies [6,51,52], education played a significant role in e-cigarette use behavior among heterosexual individuals. Those with some college education (versus less than high school education) had higher odds of being former e-cigarette users. However, no statistically significant differences in former e-cigarette use were observed between those with less than high school education and their counterparts with high school or college graduate/higher education. No statistical differences were also found in current e-cigarette use behavior in heterosexual individuals. Similarly, we did not find any statistically significant association between e-cigarette use behaviors and education among sexual minority individuals, indicating that education might play less of a role in e-cigarette use among Black/African American sexual minority individuals. In addition to education, general health status and region of residence were not significantly associated with e-cigarette use behaviors among Black/African American heterosexual and sexual minority persons. Thus, e-cigarette use among these persons might have been independent of whether they had poor or good health, or where they resided in the U.S. It is possible that similar discrimination experienced in educational attainment and healthcare might also translate into an analogous e-cigarette use behavior among this population. Minority persons, such as those with a sexual minority identity and Black/African American persons, often experience discrimination and stigmatization; therefore, the intersection between sexual identity and Black/African American race further decreases differences in their behavioral outcomes, including e-cigarette use, irrespective of their education, general health status, or wherever they may reside [45,53,54,55].

While income is an important sociodemographic determinant of e-cigarette use in the general population with a higher household income associated with lower former or current e-cigarette use [6,52,56], this association might vary within subgroups. We found that while Black/African American heterosexual individuals with higher incomes were less likely to use e-cigarettes, Black/African American sexual minority individuals with higher incomes were more likely to use e-cigarettes. A higher income was associated with lower odds of former and current e-cigarette use among heterosexual persons, whereas a higher income was only associated with higher odds of former e-cigarette use among sexual minority persons. These findings indicate that while lower odds of e-cigarette use were noted among Black/African American adults in general population studies, the intersection of sexual identity and income levels makes some Black/African American adults more, less, or just as likely to use e-cigarettes. Considering these intersected identities in future studies and interventions may provide sufficient and tailored resources to reduce tobacco use cessation disparities among Black/African American adults.

Our findings support the growing evidence that individuals who experience mental health disorder symptoms, including anxiety/depression, may be at increased risk of e-cigarette use [7,21,22,28,29]. The relationship between symptoms of anxiety/depression and e-cigarette use varied by sexual identity in our study among the Black/African American population. In particular, it is not surprising that the Black/African American sexual minority and heterosexual persons who experienced anxiety/depression symptoms were more likely to engage in e-cigarette use than their counterparts. Black/African American and sexual minority adults disproportionately have the highest burdens of anxiety/depression symptoms due to persistent discrimination, stigmatization, prejudice, and health disparities, which often trigger increased substance use (e.g., e-cigarette use) as a coping mechanism [18,19,28,50,57,58]. However, it is vital to note that the differences in current e-cigarette use behavior based on anxiety/depression symptoms were not statistically significant among the sexual minority persons. Perhaps the intersection of being Black/African American and identified as a sexual minority might have compounded the marginalization and minority stress leading to increased anxiety/depression symptoms, which might have had a similar effect on their e-cigarette use behavior.

Our study used nationally representative data to assess e-cigarette use behaviors among Black/African American adults based on their sexual identity, which is a population that has been understudied concerning e-cigarette use behaviors. However, our study had some limitations. Because we used cross-sectional data, we were unable to examine the temporal sequence of the e-cigarette use behaviors and the independent variables. Hence, we were unable to establish causality in this study. The statistical inferences were centered on the interpretation of associations. The data were also based on self-reported responses, which are susceptible to response biases and lead to underestimating behavior and statistical estimations.

## 5. Conclusions

This current study contributes to bridging the gap in e-cigarette use literature among the Black/African American adult population. The findings exhibit the heterogeneity within the Black/African American population and differences with other racial/ethnic groups (e.g., Whites), indicating the dangers of not considering subgroup differences as a standard part of public health research practice. Notably, sexual identity and anxiety/depression intersections significantly influenced e-cigarette use in different ways among this population. Our findings further imply that tobacco-use-related health disparities among the Black/African American population might have been underestimated previously. That is, sexual minority persons and those with anxiety/depression symptoms, who already have high burdens of health-related outcomes, also had a higher risk of e-cigarette use in Black/African Americans, which is the population with the highest tobacco-use health-related problems. The public health community should consider these within-group differences in tobacco control interventions aimed at reducing tobacco-use-related health disparities among Black/African American subgroups, as these populations are often targeted by the tobacco industry with tobacco products. The results also underscore the need for longitudinal data on e-cigarette use to examine the long-term mechanisms of e-cigarette use and its health effects based on sexual identity within the Black/African American populations to provide evidence of e-cigarette use disparities. Future studies may also compare e-cigarette use behaviors within racial/ethnic minority groups, especially based on their age groups (e.g., youths compared with adults), using large national data sets.

## Figures and Tables

**Table 1:** The weighted prevalence of e-cigarette use stratified by sexual identity among Black/African American adults (n = 6267) by their sociodemographic characteristics and anxiety/depression symptoms.

	Heterosexual					Sexual Minority				
	Overall Sample	Never Used E-Cigarettes	Former E-Cigarette Use	Current E-Cigarette Use		Overall Sample	Never Used E-Cigarettes	Former E-Cigarette Use	Current E-Cigarette Use	
	n (%)	n (%)	n (%)	n (%)	*p*-Value	n (%)	n (%)	n (%)	n (%)	*p*-Value
**Overall**	5820	5199 (86.16)	499 (10.19)	122 (3.65)		447	343 (70.70)	74 (23.53)	30 (5.78)	
**Age**					<0.001					0.007
18–25	187 (11.53)	148 (78.03)	29 (13.45)	10 (8.52)		34 (19.01)	26 (75.50)	7 (24.50)	1 (<0.001)	
26–34	454 (12.90)	364 (78.44)	73 (17.97)	17 (3.59)		78 (19.38)	50 (68.15)	21 (21.13)	7 (10.71)	
35–49	1301 (29.63)	1117 (85.32)	138 (10.41)	46 (4.28)		106 (26.40)	68 (47.34)	24 (42.95)	14 (9.71)	
50–64	2254 (32.03)	216 (88.43)	194 (8.77)	44 (2.79)		142 (23.37)	118 (83.51)	17 (12.53)	7 (3.97)	
65 or older	1624 (13.91)	1554 (96.64)	65 (3.07)	5 (2.92)		87 (11.85)	81 (93.94)	5 (4.28)	1 (1.77)	
**Sex**					0.004					0.256
Male	1888 (42.46)	1644 (82.47)	194 (12.55)	50 (4.98)		141 (50.65)	108 (65.78)	26 (29.35)	7 (4.87)	
Female	3932 (57.54)	3555 (88.89)	305 (8.45)	72 (2.66)		306 (49.35)	235 (75.75)	48 (17.55)	23 (6.71)	
**Level of education completed**					0.035					0.719
Less than high school	649 (15.51)	580 (87.66)	54 (9.05)	15 (3.29)		91 (25.59)	76 (72.13)	10 (23.71)	5 (4.16)	
High school graduate	1383 (27.22)	1243 (85.34)	103 (9.11)	37 (5.55)		97 (27.64)	71 (71.77)	18 (22.78)	8 (5.45)	
Some college	1795 (30.35)	1562 (84.17)	193 (13.63)	40 (2.21)		129 (29.76)	95 (63.56)	23 (26.80)	11 (9.64)	
College graduate or higher	1993 (26.92)	1814 (88.38)	149 (8.06)	30 (3.56)		130 (17.01)	101 (79.28)	23 (18.75)	6 (1.97)	
**Total family annual income**					0.090					0.071
Less than $20,000	2100 (34.37)	1826 (83.00)	222 (12.57)	52 (4.43)		220 (48.91)	166 (79.27)	34 (14.39)	20 (6.35)	
$20,000 to <$35,000	995 (16.44)	893 (85.54)	79 (9.45)	23 (5.01)		81 (13.59)	69 (73.14)	10 (25.70)	2 (1.16)	
$35,000 to <$50,000	819 (13.97)	746 (90.51)	60 (8.57)	13 (0.92)		62 (11.00)	47 (72.07)	11 (18.16)	4 (9.78)	
$50,000 to <$75,000	863 (15.50)	780 (85.39)	67 (9.79)	16 (4.82)		34 (6.94)	24 (45.08)	8 (38.57)	2 (16.35)	
$75,000 or more	1043 (19.73)	954 (89.72)	71 (8.12)	18 (2.16)		50 (19.56)	37 (55.90)	11 (42.55)	2 (1.55)	
**General health status**					0.641					0.378
Excellent/very good/good	4473 (78.99)	4033 (86.46)	352 (9.85)	88 (3.69)		328 (72.26)	255 (73.24)	56 (22.80)	17 (3.96)	
Fair or poor	1347 (21.01)	1166 (85.04)	147 (11.46)	34 (3.50)		119 (27.74)	88 (64.06)	18 (25.42)	13 (10.52)	
**U.S. census region**					0.166					0.890
Midwest	988 (16.45)	869 (86.55)	95 (7.83)	24 (5.62)		72 (16.67)	58 (62.56)	7 (29.01)	7 (8.43)	
Northeast	867 (17.71)	776 (86.13)	70 (8.71)	21 (5.17)		64 (18.71)	50 (77.87)	10 (19.27)	4 (2.86)	
West	633 (11.69)	571 (84.27)	48 (13.00)	14 (2.74)		68 (15.40)	49 (71.43)	16 (24.74)	3 (3.84)	
South	3332 (54.15)	2983 (86.47)	286 (10.79)	63 (2.75)		243 (49.22)	186 (70.50)	41 (22.91)	16 (6.59)	
**Current anxiety/depression status**					0.001					0.215
No/normal	4055 (68.91)	3719 (88.34)	275 (9.12)	61 (2.54)		201 (42.63)	165 (73.01)	29 (23.74)	7 (3.24)	
Mild	972 (16.83)	837 (84.30)	105 (10.21)	30 (5.49)		125 (27.56)	101 (74.22)	17 (21.49)	7 (4.29)	
Moderate	434 (7.26)	343 (78.37)	77 (17.99)	14 (3.64)		68 (15.69)	39 (55.16)	20 (37.81)	9 (7.03)	
Severe	359 (7.00)	300 (77.29)	42 (12.58)	17 (10.13)		53 (14.12)	38 (74.09)	8 (10.99)	7 (14.92)	

Data source: Health Information National Trends Survey. Statistically significant at *p* < 0.05 using chi-squared tests. The main variables are in bold.

**Table 2:** Weighted multinomial logistic regression analysis of factors associated with e-cigarette use behaviors among Black/African American adults who identified as heterosexual vs. sexual minority individuals.

	Heterosexual				Sexual Minority			
	Never Used E-Cigarette Versus:				Never Used E-Cigarette Versus:			
	Former E-Cigarette Use		Current E-Cigarette Use		Former E-Cigarette Use		Current E-Cigarette Use	
	RRR	95% CI	RRR	95% CI	RRR	95% CI	RRR	95% CI
**Age**								
18–34	Ref							
35–49	0.65	(0.41, 1.04)	0.69	(0.34, 1.42)	3.54 *	(1.36, 9.21)	2.60	(0.60, 11.27)
50 or older	0.38 ***	(0.24, 0.60)	0.29 **	(0.13, 0.67)	0.32 *	(0.10, 0.99)	0.49	(0.12, 2.02)
**Sex**								
Male	Ref							
Female	0.57 **	(0.40, 0.82)	0.45 **	(0.26, 0.78)	0.44	(0.17, 1.15)	1.14	(0.33, 3.87)
**Level of education completed**								
Less than high school	Ref							
High school graduate	1.20	(0.67, 2.16)	2.11	(0.83, 5.34)	0.73	(0.19, 2.89)	2.42	(0.42, 14.12)
Some college	1.90 *	(1.04, 3.50)	0.84	(0.33, 2.13)	0.63	(0.18, 2.19)	3.04	(0.65, 14.25)
College graduate or higher	1.19	(0.62, 2.27)	1.38	(0.50, 3.80)	0.29	(0.07, 1.21)	0.62	(0.07, 5.12)
**Total family annual income**								
Less than $20,000	Ref							
$20,000 to <$35,000	0.71	(0.42, 1.22)	1.17	(0.51, 2.69)	3.46	(0.80, 15.05)	0.28	(0.04, 1.92)
$35,000 to <$50,000	0.56 *	(0.32, 0.97)	0.20 **	(0.07, 0.57)	2.17	(0.64, 7.31)	2.37	(0.50, 11.25)
$50,000 or more	0.64	(0.37, 1.08)	0.75	(0.30, 1.85)	4.37 *	(1.42, 13.48)	1.88	(0.35, 10.00)
**General health status**								
Excellent/very good/good	Ref							
Fair or poor	1.22	(0.84, 1.76)	0.74	(0.38, 1.45)	1.12	(0.40, 3.19)	2.62	(0.63, 10.94)
**U.S. census region**								
Midwest	Ref							
Northeast	1.24	(0.69, 2.21)	1.08	(0.38, 3.06)	1.02	(0.22, 4.86)	0.49	(0.06, 4.01)
West	1.71	(0.90, 3.27)	0.59	(0.22, 1.56)	0.82	(0.18, 3.73)	0.29	(0.03, 2.74)
South	1.45	(0.92, 2.29)	0.56	(0.25, 1.28)	1.62	(0.49, 5.29)	0.72	(0.15, 3.55)
**Current anxiety/depression status**								
No/normal	Ref							
Mild	1.20	(0.79, 1.82)	2.89 **	(1.34, 6.27)	1.11	(0.40, 3.05)	1.34	(0.29, 6.29)
Moderate	2.10 **	(1.31, 3.38)	1.99	(0.84, 4.70)	4.53 *	(1.38, 14.82)	2.91	(0.64, 13.12)
Severe	1.39	(0.71, 2.75)	5.44 ***	(2.23, 13.23)	0.52	(0.13, 2.10)	2.79	(0.52, 15.14)

RRR—relative risk ratio. Ref.—reference group. 95% CI—95% confidence interval. * *p* < 0.05, ** *p* < 0.01, *** *p* < 0.001. The main variables are in bold.

## Data Availability

The data and materials are publicly available at https://hints.cancer.gov/data/Default.aspx (accessed on 3 May 2022).

## Supplementary Materials

The following supporting information can be downloaded from https://www.mdpi.com/article/10.3390/ijerph20032078/s1, Figure S1: The prevalence of e-cigarette use behaviors (never, former, and current user) by sexual identity (heterosexual, lesbian/gay, bisexual, and other) among Black/African American adults; Figure S2: The prevalence of e-cigarette use behaviors by anxiety/depression based on sexual identity groups among Black/African American adults; Table S1: The unadjusted association between e-cigarette use behaviors and sexual identity groups (heterosexual, lesbian, gay, bisexual, and other) among Black/African American adults.
