# Supplementary material for: Association between E-Cigarette Use Behaviors and Anxiety/Depression among Black/African American Adults Based on Sexual Identity
Source: Int J Environ Res Public Health. 2023 Jan 23;20(3):2078. doi: 10.3390/ijerph20032078 (PMC9915158; doi:10.3390/ijerph20032078)
Supplement: Supplementary file 1 [file ijerph-20-02078-s001.zip › ijerph-2095131-supplementary.pdf]

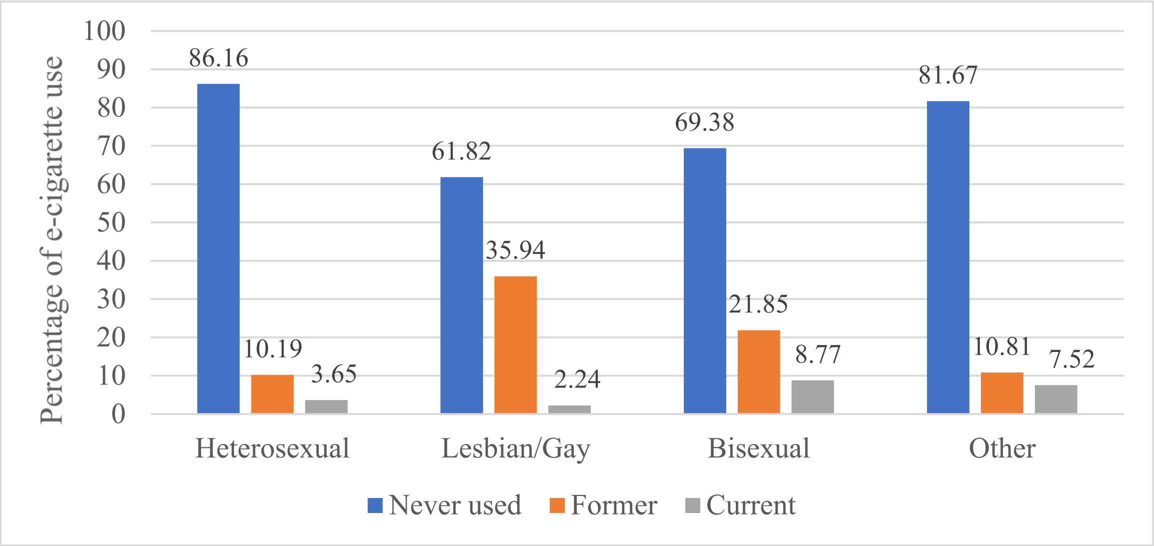

Figure S1. The prevalence of e-cigarette use behaviors (never, former, and current user) by sexual identity (heterosexual, lesbian/gay, bisexual, and other) among Black/African American adults.

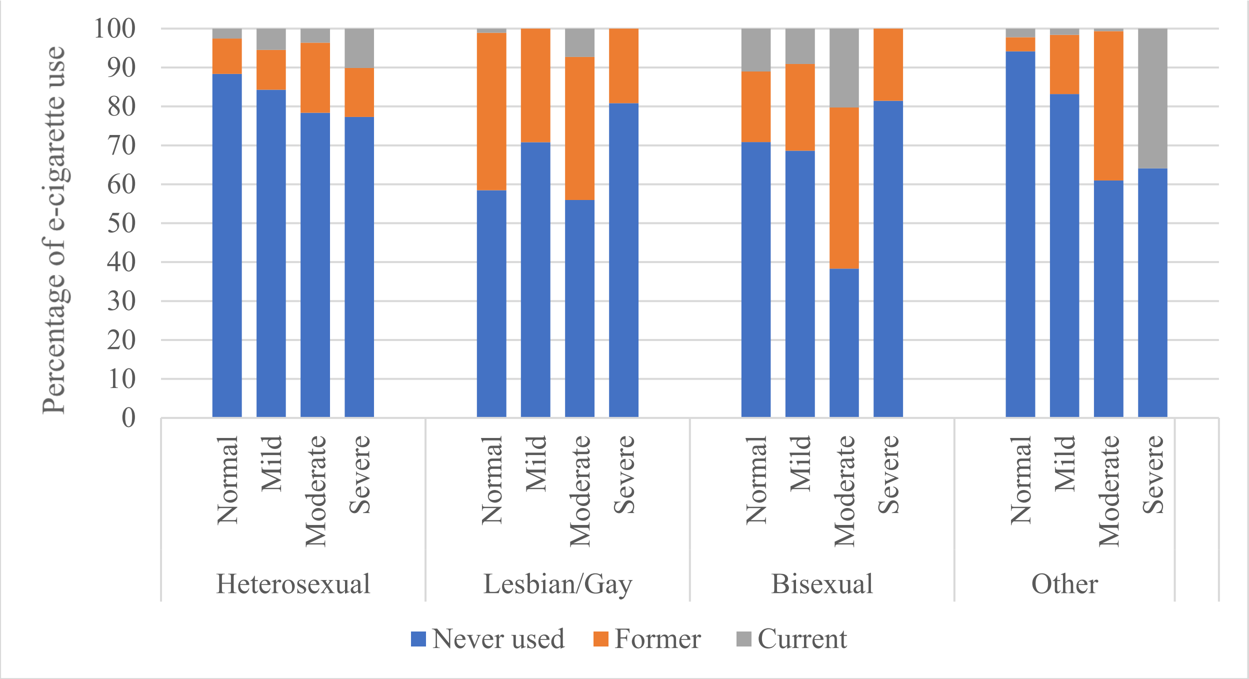

Figure S2. The prevalence of e-cigarette use behaviors by anxiety/depression based on sexual identity groups among Black/African American adults.

Table S1. The unadjusted association between e-cigarette use behaviors and sexual identity groups (heterosexual, lesbian, gay, bisexual, and other) among Black/African American adults.

	Never Used E-Cigarette Versus:			
	Former Use		Current Use	
	RRR	95% CI	RRR	95% CI
**Sexual identity**				
Heterosexual	Ref			
Lesbian/gay	4.92 ***	(2.13, 11.34)	0.86	(0.27, 2.72)
Bisexual	2.66 *	(1.12, 6.35)	2.99 *	(1.30, 6.83)
Other	1.12	(0.45, 2.77)	2.18	(0.84, 5.64)

RRR—relative risk ratio. Ref.—reference group. 95% CI—95% confidence interval. * $p < 0.05$, *** $p < 0.001$.
